# Risk factors for readmission in patients discharged with outpatient parenteral antimicrobial therapy: a retrospective cohort study

**DOI:** 10.1186/s40360-018-0240-3

**Published:** 2018-08-06

**Authors:** Victoria Huang, Jorg J. Ruhe, Polina Lerner, Marianna Fedorenko

**Affiliations:** 1 Department of Pharmacy, Mount Sinai Beth Israel, First Avenue at 16th St, New York, NY 10003 USA; 2 Division of Infectious Diseases, Mount Sinai Beth Israel, Icahn School of Medicine at Mount Sinai, First Avenue at 16th St, New York, NY 10003 USA; 3 Department of Pharmacy, Mount Sinai Hospital, 1468 Madison Ave, New York, NY 10029 USA

**Keywords:** OPAT, Risk factors, Readmission, PICC, Midline

## Abstract

**Background:**

Outpatient parenteral antimicrobial therapy (OPAT) is a practical and effective way of delivering antimicrobial therapy, but may be associated with significant risk for hospital readmission. This study aimed to elucidate risk factors related to 30-day readmissions in patients who were discharged with OPAT at Mount Sinai Beth Israel (MSBI).

**Methods:**

This IRB approved retrospective cohort study included patients who were at least 18 years or older, admitted to MSBI from August 2015 to March 2016, and discharged to receive OPAT. Patients with intravenous antibiotics prescribed for chronic suppression or planned readmission within 30 days were excluded. The main outcome was readmission to the hospital within 30 days from previous hospital discharge. Univariate and logistic regression analyses were performed to determine predictors of 30-day readmission.

**Results:**

There were a total of 200 patients included in the analysis; the median age was 60 years, 65.5% were male, and the median Charlson score was 2. A total of 155 (78%) patients received a peripherally inserted central catheter (PICC); the remainder was discharged with a midline. The most common medications prescribed for OPAT included cephalosporins (41%), vancomycin (31%), carbapenems (23%), and penicillins (16%). A total of 42 patients (21%) were readmitted within 30 days after previous discharge. Discharge to a skilled nursing facility or subacute rehabilitation center was found to be an independent predictor of readmission on logistic regression analyses (*p* <  0.05).

**Conclusion:**

Readmissions are common in patients discharged with OPAT. Recognizing predictors of readmission may help determine strategies to optimize care.

## Background

Since the 1970s, outpatient parenteral antimicrobial therapy (OPAT) has played an important role in allowing for the discharge of stable inpatients who necessitate intravenous (IV) antimicrobial therapy [1]. An estimated 1 out of 1000 Americans today receive OPAT annually, and this number is projected to grow with the continual advances in the healthcare environment and shifts in healthcare delivery. There are several advantages to OPAT, including reduced hospital length-of-stay, patient satisfaction, and potential cost savings. However, due to decreased healthcare provider supervision and environmental control, OPAT can also increase the risk for adverse drug reactions (ADR), hospital readmission, and other adverse events [1–3].

Past studies have described 30-day readmission rates ranging from 6 to 26% in patients discharged with OPAT [4–10]. This wide range may be attributed to different patient populations, indications, and study designs. Independent risk factors for readmission identified in these studies have been hypothesis generating; however, more studies are needed to explore predictors for readmission in varied institutions and patient populations. Additionally, while peripherally inserted central catheters (PICC) are historically the leading vascular access choice for OPAT, midline usage has been increasing [3]. Limited data suggest that there is comparable safety and efficacy between midlines and PICCs, but further evidence is required [11–14]. The objective of this study is to identify risk factors for 30-day readmission in patients discharged with OPAT in order to determine strategies to optimize care.

## Methods

### Study design

This study is a single-center, retrospective cohort study of patients who were admitted and discharged from Mount Sinai Beth Israel (MSBI) to receive OPAT between August 1, 2015 and March 31, 2016. Patients were identified using a report from the vascular access team of PICC and midline insertions during the study period. The Institutional Review Board (Mount Sinai School of Medicine IRB#7 – NFL – ID# IRB-16-00831) approved this study as an exempt study.

### Inclusion criteria

Patients were included based on the following criteria: 1) 18 years of age or older, 2) admitted to MSBI during the study period, and 3) discharged to receive OPAT through a PICC or midline.

### Exclusion criteria

Patients were excluded if they: 1) were prescribed IV antibiotics for chronic suppression, 2) were discharged but never received OPAT, or 3) had a planned readmission within 30 days. Furthermore, only one admission per patient was included in the study; subsequent readmissions after the index hospitalization for patients who received prior OPAT during the study period were excluded.

### Study setting

During the study period, MSBI did not have a standardized OPAT program. There are three Infectious Diseases (ID) consult groups with institutional privileges, which include a faculty teaching service and private ID groups. ID consultation is strongly encouraged by the vascular access team for all patients discharged with OPAT, but it remained at the discretion of the primary team. In regards to line selection, patients were eligible to be discharged with a midline if: the planned duration of the midline was less than 28 days from the date of insertion, the antibiotics were compatible with a midline, and the home infusion company accepted treatment with a midline.

### Data collection, definitions, and variables

Documented notes in the institution’s electronic medical record (EMR) were used to collect information on subjects’ demographics, comorbidities, hospital courses, scheduled post-discharge follow-up appointments, indications for OPAT, microbiology, antimicrobial regimens, disposition locations, vascular access types, and all ADRs and reasons for readmission. Subjects were determined to have an assigned primary care provider (PCP) if one was recorded in the EMR. Comorbidities were evaluated using the Charlson Comorbidity Index (CCI) to estimate 10-year mortality [15]. Immunosuppression was defined as a recorded history of AIDS, cancer, solid organ transplant, or bone marrow transplant. Furthermore, patients were determined to have a multi-drug resistant (MDR) organism if the EMR noted the presence of methicillin-resistant *Staphylococcus aureus* (MRSA), vancomycin-resistant enterococci (VRE), extended-spectrum beta-lactamase (ESBL) organisms, or carbapenem-resistant enterobacteriaceae (CRE). The primary clinical team (medicine or surgery) and the presence of ID consultation were also collected. Subjects were identified as having an outpatient follow-up appointment if an appointment was listed in their discharge summaries. Subjects were further analyzed on whether a follow-up appointment was planned with an ID physician.

Planned OPAT duration was defined as the number of days from the date of discharge to the planned OPAT end-date described in the patient notes. If no end-date was specified, then it was inferred based on the dosage and quantity prescribed. Readmission was defined as an unplanned hospitalization for any cause to MSBI within 30 days of the index hospitalization discharge date. This included admissions to an observation unit, but excluded admissions to the emergency department. Readmissions to other facilities were not obtainable. Readmitted subjects were identified as having a line complication if their chart notes described thrombosis, phlebitis, infiltration, extravasation, dislodgement, broken line, leakage, bleeding, or pain associated with the IV line.

### Outcome measures

The main outcome of this study was readmission to MSBI within 30 days post-discharge after using OPAT. Risk factors and predictors associated with readmission were assessed. The rate of 30-day readmission for those discharged with midlines compared to PICC lines was also evaluated.

### Statistical analysis

All variables were analyzed using descriptive and inferential statistics as appropriate using Microsoft Excel 2010 (Microsoft Corp., Redmond, WA) and IBM SPSS (version 13) software. Medians and interquartile ranges (IQRs) or ranges were reported for continuous variables. Nominal and categorical variables were compared using the χ2 or Fisher’s exact test, and continuous variables were compared using the Mann-Whitney *U* test. Univariate and logistic regression analyses were performed to determine predictors of 30-day readmission. Variables with *p* <  0.2 were included in the logistic regression.

## Results

**Fig. 1:**
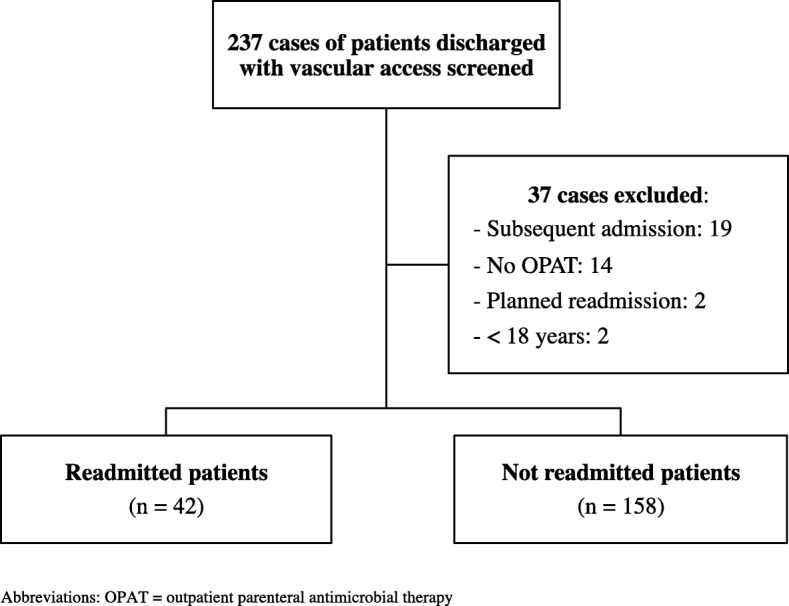
Study Population Flow Diagram

A total of 237 cases of patients discharged with vascular access during the study period were screened. Of these cases, 37 cases were excluded; a total of 200 patients were included in this study (Fig. 1). Reasons for exclusion included subsequent readmissions (19 patients), no record of OPAT (14 patients), planned readmissions (2 patients), and younger than 18 years of age (2 patients). Patient and antimicrobial characteristics are listed in Table 1. The median age was 60 years, and 65.5% were male. The majority of patients had government-funded insurance (70%) and had an assigned primary care provider (65.5%). The median CCI score was 2. Diabetes, immunosuppression, connective tissue disease, chronic pulmonary disease, and peripheral vascular disease were the most common comorbidities.

**Table 1:** Baseline Characteristics and Predictors of 30-Day Readmission in Patients Receiving Outpatient Parenteral Antimicrobial Therapy

Parameter	Total (*N* = 200)	Readmitted (*n* = 42)	Not Readmitted (*n* = 158)	Univariate analysis *p*-value
Age (years) [median (range)]	60 (20–95)	66.5 (29–95)	59 (20–92)	< 0.01
Male gender	131 (65.5)	28 (66.7)	103 (65.2)	> 0.20
Weight (kg) [median (range)]	79 (31–176)	75.5 (52–147)	80.5 (31–178)	0.08
Assigned primary care provider	131 (65.5)	27 (64.3)	104 (65.8)	> 0.20
Insurance				> 0.20
Government-funded	140 (70)	29 (69.0)	111 (70.3)	
Private	56 (28)	12 (28.6)	44 (27.8)	
Self-pay	4 (2)	1 (2.4)	3 (1.9)	
Charlson Comorbidity Index [median (IQR)]	2 (1–3)	2 (1–3)	2 (1–3)	> 0.20
Any diabetes	82 (41)	21 (50)	61 (38.6)	0.18
Immunosuppression	36 (18)	8 (19)	28 (17.7)	> 0.20
Connective tissue disease	35 (17.5)	8 (19)	27 (17.1)	> 0.20
Chronic pulmonary disease	34 (17)	5 (11.9)	29 (18.4)	> 0.20
Peripheral vascular disease	31 (15.5)	6 (14.3)	25 (15.8)	> 0.20
CVA/dementia	20 (10)	7 (16.7)	13 (8.2)	0.14
Mild liver disease	15 (7.5)	5 (11.9)	10 (6.3)	> 0.20
Congestive heart failure	13 (6.5)	5 (11.9)	8 (5.1)	0.15
Moderate/severe renal disease	8 (4)	1 (2.4)	7 (4.4)	> 0.20
Hemiplegia	7 (3.5)	0 (0)	7 (4.4)	> 0.20
Myocardial infarction	5 (2.5)	0 (0)	5 (3.2)	> 0.20
Peptic ulcer disease	2 (1)	1 (2.4)	1 (0.6)	> 0.20
Moderate/severe liver disease	2 (1)	1 (2.4)	1 (0.6)	> 0.20
Prior admission to MSBI within past 12 months	103 (51.5)	24 (57.1)	79 (50)	> 0.20
Prior OPAT within past 12 months	20 (10)	4 (9.5)	16 (10.1)	> 0.20
Hospital length-of stay [median (IQR)]	9 (6–15)	11.5 (7–15)	9 (5–15)	0.06
Inpatient service				> 0.20
Medicine	138 (69)	30 (71.4)	108 (68.4)	
Surgery	62 (31)	12 (28.6)	50 (31.6)	
ID consult	191 (95.5)	40 (95.2)	151 (95.6)	> 0.20
ID teaching service	100 (52.4)	24 (60)	76 (50.3)	> 0.20
Private ID attending	91 (47.6)	16 (40)	75 (49.7)	
ICU admission	48 (24)	13 (31.0)	35 (22.2)	> 0.20
Outpatient disposition/OPAT location				< 0.01
Home	120 (60)	16 (38.1)	104 (65.8)	
SNF/SAR	80 (40)	26 (61.9)	54 (34.2)	
Post-discharge follow up				> 0.20
ID follow up	129 (64.5)	27 (64.3)	102 (64.6)	
Non-ID follow up	71 (35.5)	15 (35.7)	56 (35.4)	
Prior history of MDR organisms	25 (12.5)	5 (11.9)	20 (12.7)	> 0.20
ESBL	10 (35.7)	1 (20)	9 (39.1)	> 0.20
MRSA	15 (53.6)	4 (80)	11 (47.8)	
VRE	3 (10.7)	0 (0)	3 (13.0)	
MDR organism isolated during admission	43 (21.5)	8 (19.0)	35 (22.2)	> 0.20
ESBL	17 (37.8)	4 (50)	13 (35.1)	> 0.20
MRSA	23 (51.1)	4 (50)	19 (51.4)	
VRE	5 (11.1)	0 (0)	5 (13.5)	
Indication for OPAT				
Osteomyelitis/septic arthritis	71 (35.5)	18 (42.9)	53 (33.5)	> 0.20
Skin and soft tissue infection	47 (23.5)	9 (21.4)	38 (24.1)	> 0.20
Genital/urinary tract infection	35 (17.5)	6 (14.3)	29 (18.4)	> 0.20
Pneumonia	20 (10)	4 (9.5)	16 (10.1)	> 0.20
Intra-abdominal infection	16 (8)	3 (7.1)	13 (8.2)	> 0.20
Bacteremia of unknown source	9 (4.5)	2 (4.8)	7 (4.4)	> 0.20
Prosthetic joint infection	6 (3)	1 (2.4)	5 (3.2)	> 0.20
Endocarditis	5 (2.5)	2 (4.8)	3 (1.9)	> 0.20
CNS infection	4 (2)	0 (0)	4 (2.5)	> 0.20
Additional oral antimicrobials	56 (28)	15 (35.7)	41 (25.9)	> 0.20
Total number of IV antimicrobials [median (IQR)]	1 (1–1)	1 (1–1)	1 (1–1)	> 0.20
Total number of all antimicrobials [median (IQR)]	1 (1–2)	1 (1–2)	1 (1–2)	> 0.20
Duration of IV antimicrobial therapy, including inpatient (days) [median (IQR)]	35.5 (15–43)	42 (18–46)	30 (14–43)	0.03
Planned duration of OPAT (days) [median (IQR)]	18 (7–34)	23 (9–34)	16 (7–33.5)	> 0.20
PICC line	155 (77.5)	36 (85.7)	119 (75.3)	0.15

All values expressed as n (%) unless otherwise noted

Abbreviations: *CNS* central nervous system, *CVA* cerebrovascular accident, *ESBL* extended-spectrum beta-lactamase, *ICU* intensive care unit, *ID* Infectious Diseases, *IQR* interquartile range, *IV* intravenous, *MDR* multidrug resistant, *MRSA* methicillin-resistant *Staphylococcus aureus*, *MSBI* Mount Sinai Beth Israel, *OPAT* outpatient parenteral antimicrobial therapy, *PICC* peripherally inserted central catheter, *SAR* subacute rehabilitation, *SD* standard deviation, *SNF* skilled nursing facility, *VRE* vancomycin-resistant enterococcus

The median hospital length-of-stay was 9 days. A total of 191 patients (95.5%) received an ID consult; of these cases, the ID teaching service was utilized in 100 patients (52.4%) and the private ID attending services were utilized in 91 patients (47.6%). A post-discharge follow-up appointment was scheduled with an ID physician in 129 patients (64.5%), while an appointment was scheduled with a non-ID physician in 71 patients (35.5%). A total of 120 patients (60%) were discharged home, while 80 patients (40%) were discharged to a skilled nursing facility (SNF) or a sub-acute rehabilitation center (SAR).

**Fig. 2:**
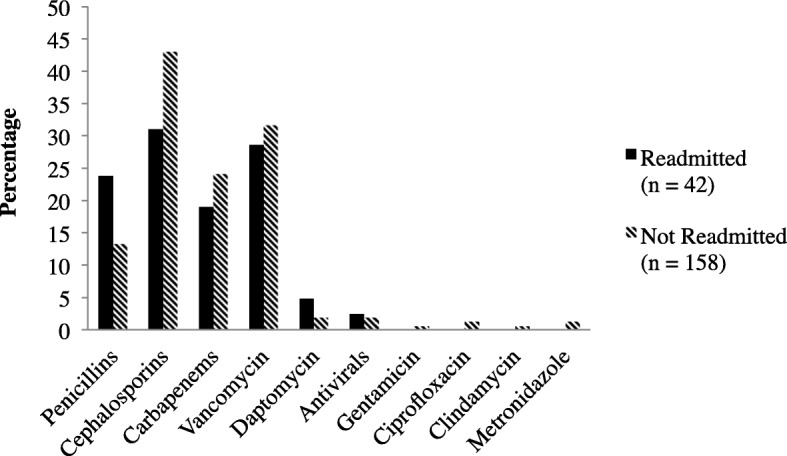
Medications Prescribed for Outpatient Parenteral Antimicrobial Therapy

The most common indications for OPAT included osteomyelitis or septic arthritis (35.5%), skin and soft tissue infection (23.5%), genital or urinary tract infection (17.5%), and pneumonia (10%). Cephalosporins (40.5%), vancomycin (31%), carbapenems (23%), and penicillins (ampicillin, ampicillin-sulbactam, nafcillin, oxacillin, penicillin G, piperacillin-tazobactam) (15.5%) were the most common antimicrobials prescribed for OPAT (Fig. 2). A breakdown of all antimicrobials prescribed is listed in Table 2. The median planned duration of OPAT was 18 days (IQR 7–34). A total of 155 patients (77.5%) were discharged to receive OPAT through a PICC line, while the remainder was discharged with a midline.

**Table 2:** Breakdown of All Antimicrobials Prescribed for Outpatient Parenteral Antimicrobial Therapy

Antimicrobial	Total (*N* = 200)	Readmitted (*n* = 42)	Not Readmitted (*n* = 158)
Penicillins	31 (15.5)	10 (23.8)	21 (13.3)
Ampicillin	4 (12.9)	1 (10)	3 (14.3)
Ampicillin/sulbactam	6 (19.3)	4 (40)	2 (9.5)
Nafcillin	5 (16.1)	0 (0)	5 (23.8)
Oxacillin	3 (9.7)	2 (20)	1 (4.76)
Penicillin G (parenteral/aqueous)	6 (19.3)	1 (10)	5 (23.8)
Piperacillin/tazobactam	7 (22.6)	2 (20)	5 (23.8)
Cephalosporins	81 (40.5)	13 (31.0)	68 (43.0)
Cefazolin	12 (14.8)	3 (23.1)	9 (13.2)
Cefepime	22 (27.2)	2 (15.4)	20 (29.4)
Ceftaroline fosamil	6 (7.4)	1 (7.7)	5 (7.4)
Ceftazidime	3 (3.7)	0 (0)	3 (4.4)
Ceftriaxone	38 (46.9)	7 (53.8)	31 (45.6)
Carbapenems^a^	46 (23)	8 (19.0)	38 (24.1)
Ertapenem	30 (65.2)	4 (50)	26 (68.4)
Imipenem/cilastatin	3 (6.5)	0 (0)	3 (7.9)
Meropenem	14 (30.4)	4 (50)	10 (26.3)
Aminoglycosides (gentamicin)	1 (0.5)	0 (0)	1 (0.6)
Fluoroquinolones (ciprofloxacin)	2 (1)	0 (0)	2 (1.3)
Clindamycin	1 (0.5)	0 (0)	1 (0.6)
Daptomycin	5 (2.5)	2 (4.8)	3 (1.9)
Metronidazole	2 (1)	0 (0)	2 (1.3)
Vancomycin	62 (31)	12 (28.6)	50 (31.6)
Antivirals	4 (2)	1 (2.4)	3 (1.9)
Acyclovir	2 (50)	0 (0)	2 (66.7)
Foscarnet	2 (50)	1 (100)	1 (33.3)

All values expressed as n (%)

^a^One patient received both meropenem and ertapenem (to start ertapenem after course of meropenem complete)

Of the 200 patients included, 42 patients (21%) were readmitted within 30 days. The median time to readmission was 11 days (IQR 5–20). Univariate analyses found that readmitted patients tended to be older (66.5 years vs. 59 years), were more likely to be discharged to a SNF or SAR (61.9% vs. 34.2%), and had a longer planned duration of IV antimicrobial therapy (42 vs. 30 days) (*p* <  0.05, respectively) (Table 1). After adjusting for confounders using logistic regression analyses, discharge to a SNF or SAR was found to be an independent predictor of readmission (adjusted OR = 3.74; 95% CI, 1.57–8.93; *p* <  0.01). Vascular access type was not found to be a risk factor for readmission.

Reasons for readmission are listed in Table 3. The most common reasons for readmission were line complications (40.5%), followed by other reasons not related to infection (28.6%). The remaining cases of readmission were due to worsening of existing infection (19%), new infection (14.3%), and ADRs (2.4%). Of the 17 patients that experienced a line complication resulting in readmission, 2 patients (11.8%) were discharged with a midline, and 15 patients (88.2%) were discharged with a PICC line. However, this difference was found to be non-significant (*p* = 1.0). Only 1 patient experienced an ADR resulting in readmission, which was found to be acute kidney injury (AKI) as a result of the usage of vancomycin dosed at 1 g IV every 12 h.

**Table 3:** Reasons for 30-Day Readmission

Reason	Readmitted^a^ (*n* = 42)
Worsening of existing infection	8 (19.0)
New infection	6 (14.3)
Not related to infection	29 (69.0)
Line complication	17 (58.6)
Other	12 (41.4)
Adverse reaction to medication^b^	1 (2.4)

All values expressed as n (%)

^a^More than one reason for readmission was noted for some patients ^b^Acute kidney injury caused by vancomycin 1 g every 12 h

## Discussion

This study observed a 21% readmission rate in patients discharged with OPAT, which is comparable to the findings of previous studies and highlights the need to improve the management and patient selection for OPAT [4–10]. Patients included in this study had a wide range of OPAT indications and comorbidities; furthermore, approximately one-quarter of the subjects were admitted to the intensive care unit (ICU).

This study identified a novel independent predictor of 30-day readmission, which was discharge to a SNF or SAR. This predictor did not overlap with what was reported in previous studies. Retrospective cohort studies of 782 OPAT patients discharged from the Tufts Medical Center and 216 OPAT patients discharged from the University of Illinois Hospital and Health Sciences System (UIHHSS) found that 26% and 20% of patients were readmitted within 30 days, respectively. Reasons for readmission included worsening infection, ADRs, line-associated complications, non-OPAT related reasons, and new infections. Independent risk factors for readmission varied and included lack of a primary care provider, previous hospital admission within the past 12 months, longer planned duration of OPAT, aminoglycoside use, bacteremia, pneumonia, history of drug-resistant organisms, and history of malignant lymphoma [4, 5].

Although the current study included significant predictors identified from these past studies as part of the analysis, these predictors were not found to be statistically significant. The disparities observed in the current research results may be due to geographical, institutional, study methodology, and sample size variations. For instance, while OPAT management at MSBI was not standardized, Tufts Medical Center had a designated clinical OPAT program, and patients who were not followed by this service were excluded. Through this program, all patients received inpatient ID consultation and were followed by an ID specialist upon discharge. Care coordination was guaranteed by an OPAT administrator who interacted with visiting nurses, outside laboratories, and outpatient infusion pharmacists to ensure regular laboratory monitoring, imaging studies, and outpatient follow-up [4]. UIHHSS did not have an OPAT program at their institution during the time of the study; however, there were dissimilarities in patient identification and selection as compared to the current study. While the current research evaluated patients discharged with both PICC lines and midlines, the UIHHSS study identified subjects using billing codes for PICC lines and IV antibiotics [5].

In the current research, prior history or isolation of a MDR organism in the index admission were not predictive of readmission, which is in contrast to the findings of the Tufts Medical Center study [4] but comparative to the UIHHSS study. Although the UIHHSS study reported that a greater proportion of readmitted patients were infected with a MDR organism compared to those that were not readmitted (40% vs. 25%, *p* = 0.054), isolation of a MDR organism was not found to be an independent risk factor for readmission in the multivariate analysis [5]. In the current research, a similar proportion of patients were identified as having a prior history or isolation of a MDR organism in the index admission in the readmission and non-readmission groups. Variability between study findings may be due to differences in study definitions of MDR organisms, patient samples, institutional settings, and other confounding factors. In addition, reasons for readmission in the subgroup of patients with MDR organisms (e.g. infection-related, line complications, or other causes) that could help clarify the association were not described. Further research is needed to evaluate management and outcomes of patients with MDR organisms that are discharged to receive OPAT.

Patients in this study who were discharged to a SNF or SAR were found to have an increased risk of readmission compared to patients who were discharged home. Although there may be more healthcare provider supervision in a SNF or SAR compared to the home setting, patients discharged to a long-term care facility may also have a higher burden of illness. This finding may therefore suggest that patients who necessitate continuity of care in a post-acute care facility after discharge may require closer observation. However, it is important to highlight that there was no significant difference between readmitted and non-readmitted patients in terms of comorbidities, CCI, or ICU admission. This may infer that factors other than disease severity play a role in readmission from a SNF or SAR. Past studies of readmissions in SNFs have found that approximately one in four patients discharged to a SNF are readmitted within 30 days [16, 17]. Many of these readmissions may potentially be avoidable, with previous studies citing communication, on-site availability of clinicians, timely laboratory tests, and adequate treatment during index hospitalization as areas of improvement [18, 19]. Implementing a process of placing follow-up phone calls to the SNF to ensure adequate continuity of care has been reported to improve outcomes in heart failure patients and possibly reduce readmissions to the hospital [20].

These data support a need for better communication, coordination, and continuity of care in patients discharged to a post-acute care facility, which could be provided by dedicated OPAT programs. The previous study at Tufts Medical Center did not find discharge to a SNF or SAR to be a risk factor for readmission; this may be related to the presence of a robust OPAT service [4]. In 2004, the Infectious Diseases Society of America provided recommendations on the fundamental elements of an OPAT program [2]. However, the prevalence of such programs is low; 26 to 56% of ID physicians reported a formal OPAT service [21, 22]. Previous studies have shown that the existence of OPAT programs with multidisciplinary teams may improve safety and efficacy outcomes, reduce 30-day readmissions, increase appropriateness of OPAT initiation, provide cost savings, and minimize adverse events [23–26].

Vascular access type was not identified as an independent factor for readmission. This observation may suggest that there is comparable safety and efficacy between PICC lines and midlines. Line complications, such as line dislodgement or infection, were the most common reasons for readmission in the current study. Of the patients that experienced a line complication, the majority was discharged with a PICC line. Limiting comparison, all subjects in the UIHHSS study and 85% of the subjects in the Tufts study had a PICC line. The remaining patients in the Tufts study had other types of vascular access that did not include midlines [4, 5]. A retrospective analysis of adult patients with cystic fibrosis who received a PICC or midline for antibiotic administration found no difference in the rate of adverse events or line removal [13]. In contrast, an observational study of 50,470 patients who received a central venous catheter for home infusion therapy found that patients with midlines had a higher incidence of total complications and catheter dysfunction but significantly less bloodstream infections as compared to patients with PICC lines [14]. These data may indicate that midlines can be a non-inferior alternative to PICC lines; however, more studies are needed to evaluate the safety and efficacy of midline versus PICC line usage in OPAT.

There was only one ADR (2.4%) observed in this study that led to a readmission, which is less than what has been reported. Previous studies have found that ADRs account for 14 to 24% of readmissions in patients with OPAT [4, 5]. This discrepancy could be due to incomplete documentation in the EMR. Patients that experience an ADR may also have followed up in the outpatient setting or were admitted to a different facility.

Strengths of this study include the breadth of patients and characteristics assessed. Although MSBI does not have a standardized OPAT service, this may have been advantageous in determining predictors of readmission in institutions without these services. The evaluation of the effects of type of vascular access on 30-day readmission is unique; findings from this study may suggest the need to further explore outcomes associated with PICC and midline usage.

There are several limitations to this study. As this was a retrospective, non-interventional study, there is an increased risk for recall and selection bias. The current research was unable to capture readmissions at other institutions. As this study utilized reports of vascular access insertion to identify patients, the current research was not able to capture patients who were discharged to receive OPAT without a midline or PICC. For instance, patients who received IV antimicrobial therapy during hemodialysis without a midline or PICC were not included in this study. Another possible source of bias is the exclusion of subsequent readmissions after the index hospitalization. The current study sample does not capture the risk factors that led to these readmissions or the outcomes that resulted from multiple readmissions. The absence of a standardized OPAT program at MSBI may have led to an increase in confounding factors, as the management of patients with OPAT may vary by clinician. However, it is important to note that over 95% of the patients studied received an ID consultation. Furthermore, outpatient records were not reviewed to evaluate clinical outcomes, availability of laboratory test results, patient adherence to scheduled follow-up appointments, whether antimicrobial regimens were changed or switched in the outpatient setting, or whether patients experienced any adverse events that necessitated follow-up. The current research was also unable to extrapolate whether specific SNFs or SARs contributed more to the readmission rate.

## Conclusion

Readmissions in patients discharged to receive OPAT were common and comparable to findings from previous studies. Discharge to a SNF or SAR was found to be a significant and novel predictor of readmission. Further research is needed to identify factors leading to increased readmission from SNFs and SARs and strategies to optimize care. While line selection did not contribute to an increased risk for readmission, the evaluation of different types of vascular access for OPAT is unique. This finding may suggest that there is comparable safety and efficacy between midlines and PICC lines for OPAT with respect to readmission. Future studies are needed to evaluate the risk factors identified and provide better recognition of predictors associated with readmission to help determine strategies to optimize care.

## Abbreviations

ADR: Adverse drug reaction
AKI: Acute kidney injury
CCI: Charlson Comorbidity Index
CNS: Central nervous system
CRE: Carbapenem-resistant enterobacteriaceae
CVA: Cerebrovascular accident
EMR: Electronic medical record
ESBL: Extended-spectrum beta-lactamase
ICU: Intensive care unit
ID: Infectious diseases
IQR: Interquartile range
IV: Intravenous
MDR: Multi-drug resistant
MRSA: Methicillin-resistant *Staphylococcus aureus*
MSBI: Mount Sinai Beth Israel
OPAT: Outpatient parenteral antimicrobial therapy
PCP: Primary care provider
PICC: Peripherally inserted central catheter
SAR: Sub-acute rehabilitation center
SD: Standard deviation
SNF: Skilled nursing facility
UIHHSS: University of Illinois Hospital and Health Sciences System
VRE: Vancomycin-resistant enterococci
